# The Treatment of Habitual Constipation

**Published:** 1894-09-01

**Authors:** 


### THE TREATMENT OF HABITUAL CONSTIPATION.

The causes of constipation are numerous and varied, but it may be generally stated that they are such conditions as produce either weakness of the peristaltic movements of the bowel, or an unnatural hardness of the intestinal contents. These two factors are frequently, indeed, usually, combined, though present in varying degree in different cases, and successful treatment largely depends on a right estimation of their relative importance in any particular instance.

By habitual constipation we may conveniently understand constipation *per se* of some duration, and it is not necessary, therefore, to enter into the condition as dependent on local lesions of symptomatic of visceral disease. It is also well to remember at the outset that a considerably longer period than usual between the evacuation of the bowel may exist in some people without impairment of health, and that persons living on a diet producing very little solid residue this state of affairs may also exist without calling for treatment.

Among the conditions leading to deficient peristaltic action a predominant place must be given to negligent habits acquired by the patient. Neglect of the calls of nature, of hurried, irregular, and often imperfect acts of defæcation lead to the overloading of the colon, and the stretching and consequent weakening of its muscular coat. At the same time the sensitiveness of the rectum so necessary to the starting of the reflex part of the act becomes blunted and aggravates the condition. Unpleasant symptoms arise, and relief is sought in the form of a purgative, with the result that immediate relief is obtained at the cost of still greater aggravation of the condition. As the case goes on anæmia occurs, produced or intensified by the constipation, and with its consequent debility adding still more to the atonic state of the intestinal canal, till the sufferer frequently settles down to regard a weekly evacuation produced by gradually-increasing doses of aperients as a natural state of affairs, or at least as one from which there is no escape. Deficient exercise involved in many sedentary occupations has also an important bearing both directly, and from the debility which often accompanies long hours in a confined atmosphere, and those persons whose work is mental suffer to a greater extent than those to whom some mechanical action is necessary.

Dietetic errors are chiefly responsible for the production of undue hardness of the fæces, though the habitual ingestion of larger quantities of food than are requisite to supply the needs of the body, tends to induce impaired intestinal action. Among errors of diet a very frequent cause is a deficiency in the consumption of water, that important constituent of the dietary table being often reduced to very low limits, and even then derived from other fluids such as tea, where the tannin materially reduces its value in liquefying the intestinal contents. Alcoholic beverages are also deficient in this respect. A dietary of which meat forms a large part or farinaceous foods predominate favours constipation. Bread when made of the finer varieties of flour from which the husk of the grain has been carefully removed loses not only much of its nutritive value, but also much of its power of stimulating the intestine.

The habitual use of very rich or indigestible articles of diet may also predispose, and the use of opiates or the slighter degrees of plumbism should not be forgotten in cases where the cause is obscure.

The result to be aimed at in the treatment of habitual constipation is to secure for the patient a natural and regular evacuation of the bowel and to render him independent of aperients as far as possible.

It is obvious therefore that any faulty habits must be corrected and a *régime* more suited to the necessities of the case introduced. The practice of going to stool daily at a definite time is of great value, and soon becomes a routine which does away with any feeling of constraint which the patient may at first experience. Brisk exercise, preferably on horseback, should also be insisted on, and the abuse of active purgatives must be stopped. The patient's dietary must also be examined, and will probably be found capable of improvement in some direction. A larger proportion of vegetables, and still more so of wholesome fruit with a restriction of meat, will in many cases be indicated. Fresh fruit might be eaten at breakfast to a greater extent than is usual in this country, while stewed fruits, such as prunes or apples, are readily obtainable, and are excellent laxatives. An increased consumption of fats or oily substances is often desirable in the form of butter, olive oil, bacon, &c. The substitution of brown bread for the finer varieties in use is a desirable alteration, and the occasional use of oatmeal (which should not be too coarse) in the form of porridge or oatcake, with the addition if need be of a little treacle, is both inexpensive and effectual. The importance of a sufficient supply of fluids has been already mentioned and should be impressed on the patient, and in addition to that consumed at meals it is often important that a tumblerful of water should be taken on rising in the morning, and in more severe cases another at night. Whether the water be hot or cold may be determined by the circumstances or the patient's choice, but it should not be merely warm, which may excite vomiting. It is best taken not at a draught but sipped at intervals during dressing.

In addition to these recommendations certain aids of a mechanical nature may be necessary, such as the occasional use of small enemata of glycerine, olive oil, or simple cold water. The last of these should be distinguished from the large distending enemas so useful for clearing out the bowel in certain cases, while here the action aimed at is tonic, with a certain degree of softening of the fæcal mass.

Another useful method is the employment of abdominal massage either by the hand or by a weight. The usual instrument employed—a heavy metal ball, which, even when padded, is hard and uncomfortable to the patient—may with advantage be replaced by an appliance recently introduced to the profession by Dr. R. W. Felkin.* This consists of a child's indiarubber ball of suitable size, nearly filled, but not distended, with swan shot, so that a certain amount of indentation is possible and the sensation of hardness is done away with, while the indiarubber (though it may be covered with advantage) is non-conducting and does not chill the patient. This ball is used by the patient himself before getting up, being rolled over the abdomen in the course of the colon for about ten minutes at a time. This method of treatment also reduces obesity, itself a factor in the production of constipation from the loss of power it causes in the action of the abdominal muscles.

In most cases when the condition is of some duration it will be necessary to resort to drugs, but it should be kept in view from the first that the object for which they are employed is not to produce purgation, but to act as tonics to the intestine in order to restore its natural function, and that they are to be discontinued when this object has been attained. Though it is now definitely ascertained that many of the more powerful aperients not only increase peristalsis but induce intestinal secretion, it is desirable to select those drugs which produce the former and trust the necessary alteration in the intestinal contents to dietetic treatment.

Among remedies of this class aloes holds a high place in the form of the well-known "dinner pill." Its action is slow, and chiefly on the colon and lower bowel. It may with advantage be combined with nux vomica or belladonna.

Cascara is also a most useful intestinal tonic when given in doses of ♏ 20-30, the official dosage of the British Pharmæcopia, ʒj.-ʒii., being much too large to obtain the most useful action of the drug, which thus prescribed acts as a purgative. It may be combined with the liquid extract of liquorice, which modifies its bitter taste and assists its action. Being a liquid preparation it has the advantage of greater facility of regulating the dose, the patient being instructed to increase or diminish it till the amount necessary to produce a natural motion is ascertained, and then to gradually reduce it till its administration may be intermitted and finally discontinued.

Saline aperients or mineral waters are less adapted for the treatment of habitual constipation than the remedies above mentioned, but the milder varieties may be given in a well-diluted form instead of plain water in the morning, the amount being reduced and plain water substituted as improvement takes place. One drug it is necessary to warn patients against—rhubarb, as its secondary astringent action renders it wholly unfitted for habitual use, and of course mercurial preparations should not be used for this purpose.

In the management of habitual constipation care and skill are frequently necessary, and though much must be left to discretion as bearing on individual peculiarities, it should be impressed on the patient that it is not the trivial complaint he often supposes, to be treated by an occasional pill, often a quack one, which leaves his last state worse than his first, and renders rational treatment, should he seek it, a much more difficult task.

* Edin. Obstet. Trans., vol. xviii., p. 229.

